# Invisible inequities in type I diabetes care in India: A multi-stakeholder qualitative study from Karnataka

**DOI:** 10.1371/journal.pgph.0005129

**Published:** 2025-09-12

**Authors:** Veruschka Pandey, Satyanarayana Ramanaik, Lalitha Krishnappa, Patel Swathe, Suresh Shastri, Santhosh Olety Sathyanarayana, Giridhara Rathnaiah Babu

**Affiliations:** 1 The International School of Bangalore (TISB), Bengaluru, Karnataka, India; 2 Karnataka Health Promotion Trust (KHPT), Bengaluru, Karnataka, India; 3 Department of Community Medicine, M.S. Ramaiah Medical College, Bengaluru, Karnataka, India; 4 Department of Community Medicine, ESIC Model Hospital & PGIMSR, Bengaluru, Karnataka, India; 5 Department of Health and Family Welfare Services, Government of Karnataka, Bengaluru, Karnataka, India; 6 Department of Paediatric and Adolescent Endocrinology, Karnataka Institute of Endocrinology and Research, Bengaluru, Karnataka, India; 7 Department of Population Medicine, College of Medicine, QU Health, Qatar University, Doha, Qatar; NYU Grossman School of Medicine: New York University School of Medicine, UNITED STATES OF AMERICA

## Abstract

Type 1 Diabetes mellitus (T1DM) affects a substantial population in India, with significant challenges related to healthcare access and financial burden. This study aims to explore the multi-level barriers and enablers of T1DM care in Karnataka state, providing evidence-based recommendations for policymakers to improve healthcare services. This qualitative study employed in-depth interviews (IDIs) and focus group discussion (FGDs) with multiple stakeholders, including ten People with Type-1 Diabetes Mellitus (PwT1DM) aged 5–23 years and their caregivers, 13 Healthcare providers (HCPs) -Endocrinologists, paediatricians, diabetes educators, and primary care physicians; and ten senior health officials and six Policymakers. Participants were selected using purposive sampling from both urban and rural settings across Karnataka. Data was collected over six months, and qualitative research software was used to analyse the transcribed data thematically. The study was approved by the Institutional Ethical Committee of M.S. Ramaiah Medical College. Significant obstacles include inadequate integration of policies within India’s national framework, erratic insulin availability, substantial out-of-pocket costs, and insufficient training for healthcare providers. Rural demographics face compounded disadvantages due to poor healthcare infrastructure, while caregivers, especially mothers, struggle with financial and emotional burdens; People with TIDM endure social stigma and mental health issues, particularly affecting females. The study highlights the critical need for formulating dedicated T1DM policies within the framework of the existing Non-Communicable Disease program. Implementation of subsidized insulin schemes and expansion of primary care services, along with a multi-sectoral strategy, encompassing enhanced training for HCPs, integration of digital health solutions, and development of community-based support systems, is essential for effective and sustainable T1DM management. Addressing financial and gender-based disparities is crucial to ensure equitable care in both urban and rural areas.

## Background

Type-1 Diabetes Mellitus (T1DM) affects approximately 8.75 million individuals worldwide, with one fifth of these occurring in low and middle-income countries (LMICs), including 17.4% affecting children and adolescents under 20 years of age [1,2]. The global prevalence of T1DM is projected to increase from 8.4 million in 2021 to 13.5 -17.4 million by 2040, reflecting a 60–170% increase. In a decade, the estimated number of children with T1DM in India increased 2.35 times to 229,400 (0–19 years) in 2021 [3–5]. The annual incidence of T1DM is 3.8 per 100,000 individuals, according to the International Diabetes Federation (IDF) Atlas [3,6]. The regional prevalence rates in India vary significantly; while Karnataka reports 17.93, Chennai and Karnal (Haryana) report 3.2 and 10.2 T1DM per 100,000, respectively [3–5].

The Karnataka Diabetes Registry (1995–2008) records various complications for persons with T1DM (PwT1DM), including severe hypoglycaemia, as well as microvascular and macrovascular issues [7]. PwT1DM often face workplace discrimination, struggle to balance health and work, have higher rates of absenteeism, and tend to retire earlier [8–10]. Additionally, T1DM causes increased adversity and distress throughout life [11], affecting life opportunities [12], marriage prospects, family planning, and social interactions due to stigma and inadequate care, especially impacting young adults and women [13]. In children with T1DM, school absenteeism and poorer academic performance are reported [14,15]. They encounter significant challenges in school, including difficulty concentrating and poor educational outcomes, which can lead to dropping out [8–10]. Also, a recent meta-analysis shows that youth with T1DM are less physically active and more sedentary than their non-diabetic peers [16]. Nearly one in five students with T1DM in India are prevented from participating in sports or excursions [10]. Such school environments and social exclusion result in long-term socioeconomic and health disadvantages for these young individuals [12].

In Karnataka, as in many parts of India, inadequate screening and delayed and ineffective management of T1DM are worsened by health systems that focus more on curative measures than on integrated, patient-centred approaches. Yet, there is a critical research gap in understanding the contextual burden, cultural expectations, and financial vulnerabilities associated with T1DM. Specifically, there is limited data examining how individuals and families navigate T1DM care in LMIC settings [7,17]. To address the research gap, we undertook a multi-level exploration of barriers and enablers to T1DM care in Karnataka. Our aim was twofold: first, to elucidate how social determinants and healthcare policies converge to impact disease management; second, to provide pragmatic recommendations for improving T1DM services within India’s evolving Non-Communicable Diseases (NCD) landscape. By highlighting these systemic and psycho-social factors, we underscore the need for reforms that extend beyond insulin provision to a more inclusive model of diabetes care, one that acknowledges local realities and bolsters support for patients and caregivers alike.

## Methodology

### Study setting and design

This study was conducted in Karnataka, India, and examined challenges and responses to T1DM through multi-stakeholder engagement. This study employed a qualitative research design, utilizing in-depth interviews (IDIs) and focus group discussions (FGDs) over six months to explore the experiences, challenges, and perspectives of key stakeholders involved in T1DM management. FGDs were conducted at clinical facilities when feasible, which encouraged dynamic exchanges revealing shared perspectives. However, due to the sensitive nature of personal challenges and emotional burdens are often less shared in groups, IDIs provided a private setting for deeper, personal sharing. While both explored similar themes, FGDs focused on shared experiences, and IDIs on individual views. No participants overlapped between the two methods.

Using purposive sampling, we aimed to include participants with diverse backgrounds and experiences. For PwT1DM and caregivers, we considered variations in age, gender, place of residence (rural or urban), and duration since diagnosis. For healthcare providers and policymakers, we included a mix of professional roles, facility types (primary, secondary, tertiary), and administrative levels (district and state). This diverse representation of participants enabled us to capture the complexity of challenges and responses related to T1DM across different settings and roles. Data collection continued until thematic saturation was achieved, which was assessed iteratively. Saturation was considered reached when no new themes emerged in at least two consecutive interviews within each participant group, and responses began to repeat. The research team jointly reviewed this process to ensure consistency and depth across stakeholder groups. Participants were recruited between November 15, 2023, and April 15, 2024, based on their availability and the convenience of scheduling.

An interview and FGD guide (S1 Appendix), developed in alignment with the study objectives, included open-ended questions to explore T1DM management strategies, personal challenges, systemic barriers, and policy gaps. FGDs were conducted only with PwT1DM and caregivers, as it was feasible to mobilize them in group settings and capture commonly shared experiences. In-depth interviews were used alongside FGDs to explore more personal or sensitive issues. For healthcare providers, policymakers, and senior officials, only in-depth interviews were conducted, as group discussions were not practical due to logistical constraints and the individualized nature of their roles.

Pilot testing was done to clarify and assess the relevance. Interviews and focus group discussions (FGDs) were conducted in Kannada, the local language, and subsequently translated into English for analysis and interpretation. Bilingual team members reviewed translations to ensure accuracy and contextual relevance. The interviews and FGDs were facilitated by trained qualitative researchers from the study team, who had no prior relationship with the participants. This helped maintain professional neutrality and reduce bias in the data collection process.

All sessions were held in private and quiet locations, such as hospital consultation rooms, health facilities, office meeting halls, or community centers, depending on participant convenience and to ensure confidentiality. Written informed consent was obtained from all participants, and sessions were audio-recorded, supplemented with field notes that captured non-verbal cues and contextual nuances. For participants who were unable to read and write, the researchers explained the research work in detail in their local language, and after clarifying their doubts, written informed consent was obtained.

The research team brought together diverse professional perspectives, including those of a public health researcher, a clinician, and an adolescent student researcher—this multidisciplinary composition enriched data interpretation by incorporating varied lenses. In many cases, shared professional or lived experiences with certain participant groups helped establish trust and rapport, facilitating more open and in-depth discussions. To complement this strength and ensure balanced perspectives, interviews and FGDs were conducted by an independent trained qualitative researcher with no prior professional relationships with participants, enabling both respectful engagement and methodological rigor. In this study, we used two types of triangulations to improve the credibility and richness of our results. First, data source triangulation involved the collection of perspectives from different stakeholder groups, including individuals with type 1 diabetes (PwT1DM), caregivers, healthcare providers, and policymakers. This approach helped us compare and verify insights from various experiences. Second, investigator triangulation was applied by team members from different disciplinary backgrounds who independently coded the data and developed themes, thereby increasing the rigor of our interpretation. (S2 Appendix)

#### Ethical considerations.

This study adhered to established ethical guidelines to ensure the protection of participant rights, confidentiality, and data integrity. Written informed consent was obtained from all adult participants before data collection. For children and adolescents under 18 years of age, assent was obtained in addition to written consent from their parents or caregivers. Participation was voluntary, and individuals had the right to withdraw at any stage without consequences.

To maintain confidentiality, all collected data were anonymized, with no personally identifiable information recorded or disclosed. Transcripts and interview notes were securely stored, and access was restricted to authorized research personnel. Ethical approval for the study was obtained from the Institutional Ethics Committee of M.S. Ramaiah Medical College, Bangalore, before the commencement of data collection (MSRMC/EC/AP-02/10–2023 dated 31^st^ October 2023), which accorded six months’ permission for the conduct of the study.

### Data analysis

All collected data, including audio recordings and field notes, were systematically organized, stored, and managed in a secure database using NVivo (version 11) qualitative data analysis software. Audio recordings, conducted in Kannada, were transcribed verbatim in the same language and later translated into English by an independent trained qualitative researcher. Transcripts were cross-checked with field notes to ensure accuracy and capture contextual nuances. Anonymization protocols were followed to remove identifying information before analysis.

We employed thematic analysis following Braun and Clarke’s (2006) framework, using a hybrid approach that combined both inductive and deductive coding [18]. Deductive codes were based on key domains, including access to care, insulin supply and affordability, health system responsiveness, and policy engagement, while inductive codes captured emerging, context-specific patterns grounded in participant narratives. The process involved data familiarization, initial coding, theme identification, and iterative refinement through team discussions. We followed the COREQ (Consolidated Criteria for Reporting Qualitative Research) guidelines to enhance transparency and ensure methodological rigor.

We employed triangulation among stakeholder groups, including PwT1DM, caregivers, healthcare providers, and policymakers, to compare and validate the emerging themes from various perspectives.

## Results

The results of a multi-level analysis of barriers and enablers influencing T1DM care in Karnataka, India, provide an in-depth examination of the systemic, social, and behavioral challenges faced by PwT1DM, Caregivers, and Healthcare Providers (HCPs). The profile of the stakeholders, ten PwT1DM, ten caregivers, 13 HCPs, ten senior health officials, and six policymakers, has been presented in Tables 1 and 2. Policymakers included: Director, Diabetes Centre/ Director, National Health Mission/ Deputy Director, NCD/ Reproductive Child Health officers

**Table 1:** Characteristics of PwT1DM and Caregivers.

Characteristic	PwT1DM (N = 10)	Caregivers (N = 10)
Age		
Less than 10	1	0
11–15	1	0
16–20	4	0
20+	4	10
Gender		
Male	6	4
Female	4	6
Locality		
Rural	3	4
Urban	7	6
Education		
Not literate	0	2
Primary (1–8)	1	2
High school (9–10)	1	3
College (11–12)	2	2
Higher Education	6	1
Employment		
Unemployed	7	0
Daily wage labourer	0	2
Government employee	0	0
Private employee	3	4
Housewife	0	4
Duration Since Diagnosis		
Less than 1 year	1	NA
2 to 5 years	1	NA
5 to 10 years	7	NA
10 + years	1	NA

NA: Not available.

**Table 2:** Characteristics of Participants who represent Health Officials, Policymakers, and Health Care Providers Involved in T1DM Management.

Senior Health Officials and Policymakers (N = 16)	
Designation	No.
District Surgeon	4
District Health Officer (DHO)	5
Academician	1
Policy makers	6
Geographic level	
State	3
District	13
Years in Role	
Less than 1 year	14
2–3 years	2
4–5 years	0
**Health Care Providers (N = 13)**	
Designation	No.
Endocrinologist	1
Diabetes Educator & Dietitian	4
Medical Officer (MO)	2
Paediatrician	3
Physician	3
Department/Institution	
Government	12
Private	1
Level	
State	5
District	8
Years in Role	
Less than 5 years	3
5–10 years	8
10 + years	2

The summary of the results is supplemented as S1 Fig. Findings are categorized into five key themes: structural and policy-level determinants, health system capacities and service delivery constraints, patient and caregiver burden, provider and health workforce perspectives, and Emerging solutions and policy implications. Each theme is examined through a lens of contextual realities, linking individual experiences to broader systemic inefficiencies and identifying pathways for improvement.

## Structural and policy-level determinants

### Limited policy integration and financial protection

Participants, particularly policymakers and health providers, reported that T1DM remains a low-priority issue within both national and state health policy frameworks. They pointed out that, while the National Programme for Prevention and Control of Non-Communicable Diseases (NPNCD) seeks to strengthen NCD care, its emphasis is largely on Type 2 Diabetes Mellitus (T2DM), leaving the distinct needs of PwT1DM unaddressed. According to participants, unlike other NCDs, there is no strong policy framework to ensure uninterrupted access to insulin and monitoring tools, partly due to the lack of structured funding for T1DM. They attributed this low prioritization to the limited visibility of T1DM cases in routine health system data and a widespread perception that it affects relatively fewer people, especially children, making it less urgent for national-level attention.

Moreover, participants noted that state-led subsidy programs for insulin and glucose monitoring supplies are available in certain regions. However, they reported inconsistent implementation across districts, citing factors such as lack of awareness among healthcare staff, irregular supply chains, and delays in fund disbursement. Some participants also expressed concern that, despite the existence of such schemes, frontline providers and patients were often unaware of them, limiting their reach and effectiveness.

### Cost barriers and regional disparities

The cost of insulin and glucose monitoring devices has emerged as one of the most pressing barriers, with families reporting that they spend 30–50% of their income on diabetes care. Frequent insulin stock-outs in public hospitals force patients to buy expensive, unregulated medicines from private pharmacies.

A caregiver from a lower-income household shared that “*the Government hospitals provide insulin only in limited stock, and there is no guarantee it will be available when we need it. We have to buy it from private pharmacies, which cost almost half of my monthly income.*”

Un-affordability leads to insulin rationing, especially in lower-income households. A participant acknowledged, *“We sometimes reduce the insulin dose to make it last longer because we can’t always afford to buy more.”* Participants also noted that such practices disrupted glycemic control and sometimes led to health complications that required emergency care.

These financial burdens are compounded by geographical disparities in healthcare access, where rural and semi-urban areas often lack specialized diabetes clinics and endocrinologists. Participants reported that PwT1DM often travel 80–100 km or more to reach tertiary hospitals, often on a monthly basis. For children, caregivers shared that this requires missing school and parents losing daily wages or taking leave from work, which adds further economic strain.

## Health system capacities and service delivery constraints

Findings from this study highlight significant health system barriers that restrict access to timely and high-quality care for PwT1DM in India.

### Insufficient insulin supply and diagnostic services

The irregular supply of insulin in government hospitals, particularly in rural and semi-urban areas, remains a persistent challenge, forcing PwT1DM to turn to private pharmacies, which are often expensive. This financial burden results in irregular insulin use and poor glycaemia control, increasing the risk of diabetes-related emergencies such as DKA.

A district hospital physician noted that *“We receive insulin in unpredictable batches. When supplies run out, PwT1DM has to buy them from outside, which many cannot afford. We need a better system to ensure a continuous and predictable stock of insulin.”*

Many PwT1DM receive HbA1c tests and continuous glucose monitoring devices, glucose testing only during emergency visits, rather than as part of routine diabetes management, increasing the likelihood of hospitalizations. The use of regular HbA1c tests and continuous monitoring devices is often a rare phenomenon due to financial constraints and a lack of awareness. These are often tested on patients only during emergency situations and are not part of regular monitoring activity.

A government health official highlighted that “*We focus on immediate treatment rather than long-term management. Without regular monitoring tools like HbA1c tests, we are treating diabetes blindly, which leads to complications*.”

### rural-urban disparities and health infrastructure gaps

The geographical disparity in diabetes care is another major health system constraint, particularly for rural populations, where specialized diabetes clinics and endocrinologists’ care are extremely limited. Associated transportation costs, wage loss, and logistical challenges lead to missed appointments and inconsistent treatment.

A mother from a remote village shared her frustration that *“For fever or infections, we visit the local clinic, but for my child’s diabetes, we must travel to the city. The cost of transport is almost as much as the medicine itself. Sometimes we skip appointments because we just can’t afford to go.”* Another caregiver echoed this concern: *“My husband has to miss work to accompany us to the hospital. That means two days without wages. We try to combine visits, but that’s not always possible with diabetes.”* These constraints often result in delays in insulin administration, missed follow-ups, and irregular glucose monitoring. A young girl with T1DM added: *“We wait until it’s really bad before going to the city. Otherwise, it’s just too expensive and tiring.”*

A Doctor working in a rural PHC acknowledged that *“We see very few TIDM cases, and most of us are more familiar with type 2 diabetes mellitus (T2DM). Without endocrinologists, we do our best, but sometimes, by the time a child reaches a specialised centre, their condition has worsened.”*

## PwT1DM and caregiver burden

Managing T1DM is not just a medical challenge. It deeply affects families’ economic stability, emotional well-being, and social inclusion. The chronic nature of the disease, high treatment costs, and daily self-management demands place significant burdens on PwT1DM and caregivers. This burden is particularly pronounced among lower-income families and women, who face financial hardships, gender-based discrimination, and social stigma that impact access to care.

### Economic and psychological strain on families

Families often must prioritize medical expenses over other household needs, affecting the education and well-being of non-diabetic siblings. Many caregivers, particularly mothers, are forced to quit their jobs or reduce their working hours to provide full-time care.

A mother of a T1DM adolescent shared that *“I had to leave my job because my son needs constant care. Now, we are struggling financially, and sometimes I worry about whether we will have enough money to buy his next insulin dose.”*

A parent shared that *“The diagnosis was shocking. It took us a while to accept that our daughter would need daily insulin for the rest of her life. We had to quickly learn how to administer insulin and plan meals around her condition.”*

The psychological burden of T1DM extends to children and adolescents, who frequently experience anxiety, self-blame, and social isolation due to their dietary restrictions and dependence on insulin. A 14-year-old boy with T1DM stated, “I *feel guilty every time my parents struggle to buy my medicines. Sometimes, I think it would be easier if I weren’t sick.”*

The financial and emotional burden on families emphasizes the critical need for systematic interventions, such as incorporating social protection schemes, to ensure accessible and affordable T1DM care.

### Gendered barriers and social stigma

A girl shared that *“My family told me not to talk about my condition outside because they were afraid that I wouldn’t find a husband.”* Even when she needed medical care, her family hesitated to take her to the hospital. This reflected a broader pattern among families, particularly in rural and traditional settings, where concern around marital prospects led to silence or secrecy about girls’ health conditions.

These gendered restrictive norms limit the educational and career opportunities, either discouraging them from pursuing higher studies or a job.

Another PwT1DM expressed that *“I wanted to study further, but my parents said managing diabetes would be too difficult if I moved to another city for college.”*

Families often prioritize the medical needs of male members over girls, leading to inadequate or delayed care. A caregiver admitted that *“We try to manage her condition at home because taking her to the doctor frequently is difficult. We have to think about our son’s future, too.”*

These gendered barriers have severe implications for girls’ reproductive health, mental well-being, and long-term independence, especially in traditional and rural communities. These social stigma leads to isolation, bullying, and discrimination for PwT1DM in school and workplaces.

A 19-year-old college student with T1DM noted that *“During exams, I needed to take breaks to check my sugar levels, but my teachers thought I was making excuses. I stopped asking for help because I didn’t want to be treated differently.”* The impact of mental health on discrimination and social stigma is profound, with studies showing that adolescents with T1DM exhibit a twofold elevated probability of developing anxiety and depressive disorders compared to their non-diabetic peers.

## Provider and health workforce perspectives

Knowledge and Capacity Gaps: Health Care professionals (HCPs) play a critical role in the diagnosis and management of T1DM; nevertheless, systemic challenges, including insufficient training, misdiagnosis, lack of standardized treatment protocols, and workforce deficiencies, hinder their ability to provide qualitative, patient-centered care. These challenges are particularly pronounced in resource-constrained ecosystems, such as rural and semi-urban areas of India, where specialist services are not readily available at the Primary Health Centre (PHC) level.

### Limited training and misdiagnosis risks

One of the issues in medical education curricula is the inadequate emphasis on T1DM. While T2DM is extensively covered in medical curricula due to its high prevalence in India, T1DM is under considered, which leaves the General practitioners (GPs) and primary-care physicians in the country under trained and ill-equipped to diagnose and treat T1DM cases accurately.

A Physician from a government district hospital admitted that *“In medical school, we learned a lot about T2DM, but T1DM was barely covered. Many of us rely on general knowledge, which may not always be enough. This may lead to misdiagnoses, especially in young children.”*

Consequently, this knowledge gap may lead to inappropriate use of oral medication in place of insulin, which may result in late initiation of correct treatment. In addition, a lack of standard treatment guidelines may result in poor glycaemia control and trigger complications such as DKA, which may add to the financial burden on families. Also, early symptoms of T1DM, like increased thirst, frequent urination, and weight loss, often mimic Tuberculosis and urinary tract infections in regions where these are of high prevalence.

### Gaps in PwT1DM counselling and long-term care

Even if an accurate diagnosis is made, the provision of comprehensive, patient-centric T1DM care is hindered by gaps within the healthcare system. High outpatient loads, a workforce shortage, and time constraints hinder doctors in providing comprehensive care. Poor diabetes counselling services impact treatment compliance and long-term outcomes.

A physician at a government hospital highlighted that *“Most of the time, we prescribe insulin and move on to the next patient. We won’t have time to provide detailed counselling on diet and lifestyle. Many patients find out for themselves on their own.”*

Lack of adequate counselling often leads to poor compliance, with many failing to take insulin consistently due to misconceptions, fear of injections, and cost issues.

Provider noted that *“Many patients believe insulin injections are the last option and hence hesitate to start therapy.”*

This hesitation is compounded by the family’s limited understanding of the dietary modifications necessary for maintaining stable of blood sugar levels. A mother expressed: “*We don’t know which food is safe for our child.”* This is underscored by the importance of diabetes education and counselling to enhance patient outcomes.

### Shortage of diabetes educators and lack of infrastructure for PwT1DM support

In India, most Government hospitals lack a dedicated diabetes educator, even at tertiary hospitals. A practising endocrinologist from a private tertiary hospital stated that *“In urban centres, patients have access to dieticians, diabetes educators, and multidisciplinary teams. However, in rural areas, primary care doctors and nurses are often expected to perform a wide range of tasks without additional training.”*

Nurses and health workers often lack formal training in diabetes counselling, leaving them ill-equipped to provide guidance for PwT1DM. Hence, PwT1DM are left with a choice to rely on informal sources of information, which may lead to misinformation or an approach to alternative treatment

A HCP confirmed that *“Many patients receive advice from unverified sources, which leads to confusion and improper management of their condition.”*

With this unreliable information, often PwT1DM end up in hospitals during emergencies rather than engaging in regular monitoring and guidance. A medical officer noted that *“We often see diabetes patients coming with complications, as they don’t attend regular health checkups.”* These issues underline the need for structured diabetes education and periodic follow-up at the primary healthcare level to improve patient outcomes.

### Emotional and psychological burden on healthcare providers

Apart from structural limitations, HCPs also experience significant emotional stress in managing T1DM cases. Physicians working at government hospitals and in rural areas often feel frustrated and helpless while treating young PwTIDM who struggle with insulin affordability, family support, and emotional issues.

A paediatric endocrinologist from a private medical college shared that *“We often see children come with severe complications because they can’t afford insulin or didn’t understand the importance of daily injections. It’s heartbreaking to see such situations.”*

Many physicians express their helplessness to help financially disadvantaged families who can’t afford insulin. A Government doctor shared, *“We see families struggling to afford insulin, but there is little we can do beyond prescribing it. There are no subsidies or support programs in place.”*

Additionally, HCPs have a dual burden to address both medical and psychological issues of PwT1DM, as mental health services are lacking. A physician noted that *“T1DM individuals come to us not only for diabetes management but also for anxiety and depression issues. We are not trained mental health professionals, but we have no choice but to counsel them.”*

With high patient loads at government hospitals, a single doctor seeing up to 100 patients per day may lead to severe burnout.

A HCP expressed that, “*We are overwhelmed. We hardly have time to explain insulin use properly, let alone the emotional stress these families face.”* These issues underscore the need for a financial support program, integrated mental health services, and workforce expansion to mitigate HCP burnout and enhance patient care.

## Stakeholders’ perspectives on equity, sustainability, and solutions for T1DM care

Multilevel barriers to T1DM care in India need to be addressed through a multi-pronged, equity-focused approach that integrates policy, technological innovations for health system strengthening, and community-based support. Sustainable interventions addressing affordability and accessibility throughout the continuum of care should be explored, especially for marginalized populations affected by healthcare inequities. This section examines the key emerging solutions for enhancing T1DM care and improving long-term outcomes.

### Perspectives on state-subsidized insulin programs

One of the most impactful and immediate solutions is the implementation of state-subsidized insulin programs to reduce the financial burden on families. According to a few respondents, some Indian states have piloted such initiatives by ensuring the availability of free or subsidised insulin in public hospitals. While these efforts reflect an attempt toward universal access, participants noted that they remain limited in both scope and consistency of implementation across the states.

A policymaker emphasised the importance of scaling such initiatives at the national level, stating, *“We have seen successful state-level programs making insulin affordable, but we need a national-level strategy to ensure uniform access.”*

Participants suggested that a nationally coordinated insulin subsidy could be integrated into Ayushman Bharat, a public health insurance scheme, ensuring PwT1DM consistent access to insulin, especially from lower-income backgrounds. They also recommended regulating private sector insulin pricing to prevent excessive mark-ups that make insulin unaffordable for many.

### Stakeholder perspectives on gaps in primary healthcare capacity for T1DM

This study highlights the urgent need to expand the capacity of PHCs and district hospitals to provide comprehensive care for T1DM. According to respondents, most PHCs lack trained endocrinologists or medical officers who are adequately trained in T1DM, making diabetes care primarily inaccessible to rural populations. One policy stakeholder noted, *“We cannot expect rural patients to travel long distances to tertiary hospitals for routine diabetes care. Strengthening PHCs is the only way to ensure continuity of treatment”.* A healthcare administrator highlighted that *“We have seen how CHWs have been instrumental in TB and HIV programs. A similar approach in diabetes care could reduce the burden on specialists and improve patient follow-up.”*

These views reflect a shared belief among respondents that the current health infrastructure is inadequate and that decentralising diabetes care through PHC-level capacity building may help address service gaps.

### Stakeholder perspectives on digital health tools for enhancing T1DM care

Participants across stakeholder groups shared mixed views on the role of digital health tools in addressing care gaps for T1DM, especially in rural and underserved communities. Some healthcare providers have noted that mobile health (mHealth) applications and telemedicine platforms can support routine monitoring and improve access to specialists. One healthcare professional stated, *“Telemedicine can help bridge the gap for patients in remote areas who struggle to access specialised care.”*

Other suggestions included the use of automated reminders for insulin doses and digital health education modules to enhance adherence and self-monitoring, especially among adolescents. However, these ideas were accompanied by concern around feasibility.

A policy stakeholder highlighted that *“While digital tools have great potential, we need better training and support systems for both patients and HCPs to ensure effective implementation”.* Participants also identified challenges, including inadequate digital infrastructure in rural areas, low smartphone ownership, and the additional cost burden that may arise from mobile data usage or accessing telehealth platforms. These findings suggest a balance of optimism and caution in adopting digital interventions, with stakeholders emphasizing the need for low-cost, user-friendly platforms that are tailored to the realities of low-resource settings.

### Stakeholder views on policy integration: embedding T1DM in national health programs

This study highlights a critical policy gap in India’s healthcare framework, as T1DM remains largely unaddressed in the NPNCD, unlike T2DM.

A policy stakeholder noted that *“T1DM is often overlooked in national programs, leaving patients without essential support for insulin access and regular monitoring.”*

HCPs emphasized the need for the expansion of NPNCD to explicitly include T1DM-specific care, with a dedicated budgetary allocation for insulin and glucose monitoring services in public health facilities.

One caregiver, expressing frustration over the financial burden, said, *“We have to pay out of pocket for every test and insulin refill. If these were covered under public health schemes, it would alleviate many of our struggles*”.

Additionally, the study revealed the gendered impact of T1DM, with adolescent girls facing unique social barriers related to marriage, education, and stigma.

An HCP emphasized the importance of policy interventions that address these disparities, stating that “Girls with T1DM are often discouraged from higher education or marriage.” Policy changes must consider the specific needs of individuals to ensure equitable access to care. These findings highlight the urgent need for comprehensive policy reforms that integrate T1DM into NPNCD, addressing both medical and socio-economic challenges faced.

### Perspectives on community-based and peer-support diabetes management

This study highlights the potential of community-based interventions in improving self-care practices, reducing stigma, and providing psychosocial support for PwT1DM. Participants emphasised the importance of peer support groups, where PwT1DM and their caregivers share experiences and strategies for disease management.

A young participant shared that *“Talking to others who have T1DM helped me realise I’m not alone.”* I learned how to manage my insulin more effectively and cope with stress. Caregivers also found these groups beneficial, as one parent noted, *“Before joining, we were overwhelmed and confused, but now we feel more confident in managing our child’s condition.”*

The study revealed that peer-led interventions improved emotional well-being and treatment adherence by fostering a sense of belonging and shared understanding. A healthcare professional (HCP) highlighted the significance of such initiatives, stating, “*When PwT1DM see others successfully managing T1DM, it motivates them to adhere to treatment and take control of their health*.” These findings underscore the need to expand peer-led support models across India to mitigate the psychological burden of T1DM and foster community resilience.

### Stakeholder-specific solutions framework for T1DM management in karnataka

Findings from this study underscore the need for a multi-tiered approach to address the challenges in T1DM management effectively. In response, a stakeholder-specific framework has been developed, providing a clear delineation of roles across the health system, providers, and community stakeholders. This framework emphasises policy integration, strengthening the healthcare workforce, and community engagement, ensuring a sustainable and patient-centred response to T1DM. (Table 3) This structured framework bridges systemic gaps by linking challenges with actionable interventions tailored to each stakeholder. By ensuring coordinated efforts across policy, healthcare delivery, and community-level engagement, this approach enhances the accessibility, affordability, and quality of T1DM care in Karnataka.

**Table 3:** Suggested stakeholder-specific solutions framework for T1DM in Karnataka.

Challenge	Health System Response	Healthcare Provider Action	Community & Patient Engagement
**Delayed Screening & Diagnosis**	Integrate T1DM screening into Rashtriya Bal Swasthya Karyakram (RBSK)	Train PHC doctors to recognise early symptoms of T1DM	Awareness campaigns through ASHAs and schools
**Lack of Policy Integration**	Include T1DM in the National Program-NPNCD	Develop standard guidelines for paediatric diabetes care	Advocacy by PwT1DM organisations and NGOs
**Insulin Supply Chain Issues**	Ensure uninterrupted insulin supply at PHCs and district hospitals	Improve cold chain logistics for insulin storage	PwT1DM advocacy for regular supply monitoring
**Provider Training Gaps**	Incorporate T1DM modules in medical curricula	Conduct CME programs for PHC and general physicians	PwT1DM education on self-monitoring and adherence
**Financial Burden & Insurance Gaps**	Expand insurance coverage for insulin and monitoring devices	Guide PwT1DM on available government subsidies	Empower PwT1DM to seek financial aid through NGOs
**Low Counselling & Support Services**	Deploy diabetes educators at district hospitals	Train nurses & counsellors in diabetes education	Peer support groups for PwT1DM, including adolescents
**Stigma & Social Misconceptions**	Nationwide awareness campaigns targeting rural areas	Encourage schools to accommodate children with T1DM	Family-level counselling to reduce stigma
**Limited Access to Endocrinologists**	Strengthen telemedicine networks for remote consultations	Implement e-consultation models for PHCs	Utilise mobile health apps for virtual follow-ups

## Discussion

Our qualitative study in Karnataka comprehensively analyses the patient and caregiver perspectives, descriptions against the backdrop of mechanisms involved in managing the burden of T1DM in India. The results highlight that T1DM is a silent burden affecting families, yet it is obscured by policy and implementation frameworks despite its profound clinical implications [15]. Our research aligns with existing evidence that highlights fragmented health systems, inadequate policy focus, and significant budgetary strain, thereby exacerbating inequities in healthcare services for PwT1DM [19–21]. The shared realities, as reported by research participants—PwT1DMs, caregivers, HCPs, and policymakers —show that T1DM, as a medical illness, reflects only the tip of the iceberg of a rather profound socioeconomic and psychological burden on families and the healthcare system [22–24].

We have identified that inadequate policy integration and funding hinder the care of PwT1DM. While Karnataka’s health officials are eager to promote initiatives for T1DM, the lack of a cohesive national strategy prioritising this issue has led to several inconsistencies in accessing insulin, diagnostic services, and patient education. India’s NCD program, launched in 2008, is adult-centric and excludes T1DM. The reliance on national mandates to implement health programmes has historically challenged the health system in numerous states, despite health being primarily a state responsibility [25,26]. Only in recent years have NCDs begun to receive the attention they merit, due to ongoing review and guidance from the public health community [27,28]. Hence, it would be premature to assert that other NCD programmes overshadow T1DM care, particularly when they grapple with significant challenges such as inadequate screening and coverage gaps. The recurrent insulin shortages and the strain of high out-of-pocket costs highlight the urgent necessity to incorporate T1DM into NPNCD and publicly funded health insurance schemes, potentially drawing lessons from successful government-funded initiatives for other chronic diseases [27,29]. Efforts to integrate regulated pricing for insulin and manage the supply chain effectively can help alleviate the burdens faced by families. Furthermore, bolstering essential diagnostic services and reimbursing out-of-pocket expenses can help mitigate daily wage losses, thereby advancing the stated objective of universal health coverage [30].

In India, 38% of Indian households with diabetic members’ experience catastrophic health expenditure, and approximately 10% of DM‐affected households are pushed below the poverty line because of Out of pocket expenditure (OOPE). The financial hardships faced by PwT1DM often result in health costs exceeding the threshold of 10% of household consumption and greater than 40% of non-subsistence income [31]. More than 30% of families with T1DM children spend over half their income on health care; such spendings are often done through distressed financing, pushing households into poverty and highlighting the extreme vulnerability of managing chronic diseases in low-resource settings [32–34].

Our research shows how health system weaknesses and service delivery challenges extend beyond material shortages [35]. The gap between urban and rural regions, along with the scarcity of trained specialists and diabetes educators, significantly hampers the continuity of care. Rural caregivers face substantial logistical and financial challenges in accessing tertiary centres for routine check-ups and managing urgent complications. This distance disrupts timely and consistent care delivery, increasing the risk of severe complications. Additionally, primary care providers often point out their inadequate training related to T1DM, revealing that gaps in knowledge and lack of familiarity with paediatric diabetes management can lead to misdiagnoses, unnecessary delays, and inappropriate treatments like oral medications. Addressing these capacity issues is essential for reducing preventable hospitalisations and fostering early, precise management tactics.

Our findings reveal that families, particularly mothers, face significant burdens due to social stigma, gender inequities, and economic vulnerability [36]. Mothers often navigate uncertainty and anxiety while balancing strict insulin schedules and dietary plans with domestic responsibilities and financial constraints [37]. Adolescents themselves confront psychosocial challenges, such as bullying at school, social isolation, and discrimination in marriage or employment, all of which can undermine adherence and quality of life [38]. In more conservative settings, the stigma associated with chronic illness can particularly marginalise girls, limiting their educational and personal development [39,40]. Furthermore, patriarchal norms and gender-related stigma surrounding marriageability promote concealment and delay in the treatment of T1DM among adolescent girls, which contribute to emotional distress and negative health outcomes [13]. Although rarely addressed in clinical treatment protocols, these social determinants shape disease progression and outcomes.

In addition to the medical complications, T1DM imposes several socioeconomic challenges on the families, disrupting education and leading to caregiver stress and financial insecurity. Our results show that families faced significant geographic disadvantage, often having to travel long distances to seek T1DM care. This aligns with evidence indicating that travel costs and time are substantial burdens in diabetes management. These trips frequently cause children to miss school and parents to lose work hours, which can impact household income [41]. Often, indirect costs like lost wages are significant and impact many caregivers who forgo earnings to take their child to the clinic [42]. Evidence indicates that families sell assets or cut expenses, such as siblings’ education, to afford insulin and supplies [33]. Our findings on insulin rationing and delayed care-seeking predict severe medical consequences, as supported by the literature [43]. These results align with other evidence indicating inequities in T1DM care in rural primary healthcare settings [44]. Consequently, delayed referrals and complications affect PwT1DM [45]. Inadequate insulin leads to acute metabolic crises; even short lapses can cause life-threatening diabetic ketoacidosis (DKA) [46,47]. Many patients in resource-limited settings experience recurrent DKA or early death when insulin is unaffordable [48]. PwT1DM in LMICs are at the risk of premature complications and preventable mortality due to limited access to quality healthcare and the extended periods of high glucose levels. A survey in Odisha revealed that only 20% of health centers have a functioning glucometer, forcing patients to travel far for diagnostics [49]. The complications in the eyes, kidneys, and nerves require advanced care, which again is a limitation in these settings [50–52]. For example, 37% of the children with T1DM presented with Diabetic Ketoacidosis when they presented to the health system, and this was significantly associated with lower per-capita income and treatment gap [53]. Maintaining healthy blood glucose levels by reducing treatment delays and enhancing insulin access can minimize life-threatening complications and mortality [54]. Consequently, any strategy to improve T1DM care must be culturally sensitive, incorporating medical care, psychological, and social support into standard clinical practice and community outreach efforts [55]. Participants from various stakeholder groups identified promising avenues for policy and programme innovation. Firstly, scaling up state-subsidized insulin programmes and price regulation measures would substantially alleviate the financial burden on caregivers and enhance adherence. Secondly, a task-shifting model—where primary health workers, nurses, and accredited social health activists (ASHAs) receive targeted training — could aid in decentralizing T1DM care and reducing dependence on overstretched tertiary centers. Thirdly, digital health interventions can provide tele-consultation and monitoring services for T1DM, especially in remote areas. While user training and infrastructure concerns persist, well-designed mobile applications and virtual follow-up systems can bridge knowledge gaps, facilitate dose adjustments, and connect PwT1DM with specialist care without excessive travel costs. Lastly, the emotional strain on both families and frontline providers underscores the need to more concretely integrate mental health services and peer-support networks into diabetes programmes. The success of community-based group models, where PwT1DMs and caregivers share knowledge and coping strategies, illustrates the power of collective empowerment in alleviating stigma and burnout.

Youth in health programs should be viewed as more than mere beneficiaries and instead should be engaged as equal and integral partners in projects and research. This is endorsed by best practices in research as well as the Global Consensus Statement on Meaningful Adolescent and Youth Engagement [56]. The engagement of youth helps ensure that interventions are developmentally appropriate and grounded in the real challenges youths face in low-resource settings [57–60]. For instance, balancing T1DM management with school, as documented in LMIC contexts, is well contextualized by youth participation [61,62]. The student’s involvement in the study fostered empathy, seen as a hopeful shift towards youth participation in health research. Engaging students in our study promoted inclusion; the co-creation of knowledge with researchers and policymakers strengthens the trust needed in qualitative inquiry. Case studies from several LMICs show that youth-led research led to feasible, contextually specific, age-appropriate, and culturally relevant interventions [57,61–65]. Early involvement enhances the impact and expands the influence of youth on health research, relating to their lived experiences and those of their peers, and promotes their participation in health programs [63,65,66]. In systems that overlook young voices, youth-led inquiry serves as a form of advocacy, involving listening, learning, and voicing hopes aloud. Also, it fosters optimism among policymakers and clinicians [67,68]. Our study emphasizes the significance of youth engagement in research and health programs, aligning with global efforts to ensure adolescents’ rights to participate in decisions that affect them [69].

There were some limitations in our study, as the likelihood of PwT1DM accessing care from the alternative system of medicine and those on irregular medical care would have been missed, since our participants were those seeking allopathic treatment. The barriers and challenges of these could be more diversified, which would shed more light. While structural issues were emphasised, individual-level behavioural aspects affecting T1DM management, such as self-care practices, were less explored. Policymakers included in the study had limited direct experience with T1DM, which may have affected the depth of policy-related insights. While the representation of PwT1DM from urban and rural areas was addressed, the representation of PwT1DM from tribal areas was not included. However, given the situation of tribal areas, similar to challenges faced in rural areas, the results would not be grossly different and could not have greatly impacted the findings of our study. Despite these limitations, the rigorous methodology enables this study to be conceptually transferable.

## Conclusion

These findings underscore the pressing need for context-specific, evidence-based, and equity-driven reforms in integrating T1DM management into state and national programs. The neglect of care for PwT1DM should be reversed by inclusion in the NPNCD. Our findings suggest that strengthening primary healthcare systems for NCD care can ensure sustained support for PwT1DM and their caregivers. Our study indicates that contextually specific, tailored interventions-particularly those addressing issues related to stigma, gender, and economic hardship that are necessary to establish a robust system capable of managing the lifelong challenges of T1DM. By prioritising the voices of those most affected, policymakers and healthcare leaders can develop a holistic model of T1DM care that not only minimises clinical complications but also alleviates the significant emotional and financial burden on families. The successful implementation of such strategies in Karnataka may serve as a model for other regions facing similar structural and social challenges, thereby making a meaningful contribution to global efforts toward equitable diabetes care.

## Supporting information

S1 Fig Summary of the results from Multi-Level Exploration of Barriers and Enablers in Type 1 Diabetes Care in Karnataka, India. (TIFF)

S1 Appendix Guide for IDI and FGD. (DOCX)

S2 Appendix Codebook. (DOCX)

S1 Text Excerpts from qualitative transcripts. (DOCX)

S1 Checklist Inclusivity in global research questionnaire. (DOCX)
