# Supplementary figures and images for: Invisible inequities in type I diabetes care in India: A multi-stakeholder qualitative study from Karnataka
Source: PLOS Glob Public Health. 2025 Sep 12;5(9):e0005129. doi: 10.1371/journal.pgph.0005129 (PMC12431490; doi:10.1371/journal.pgph.0005129)

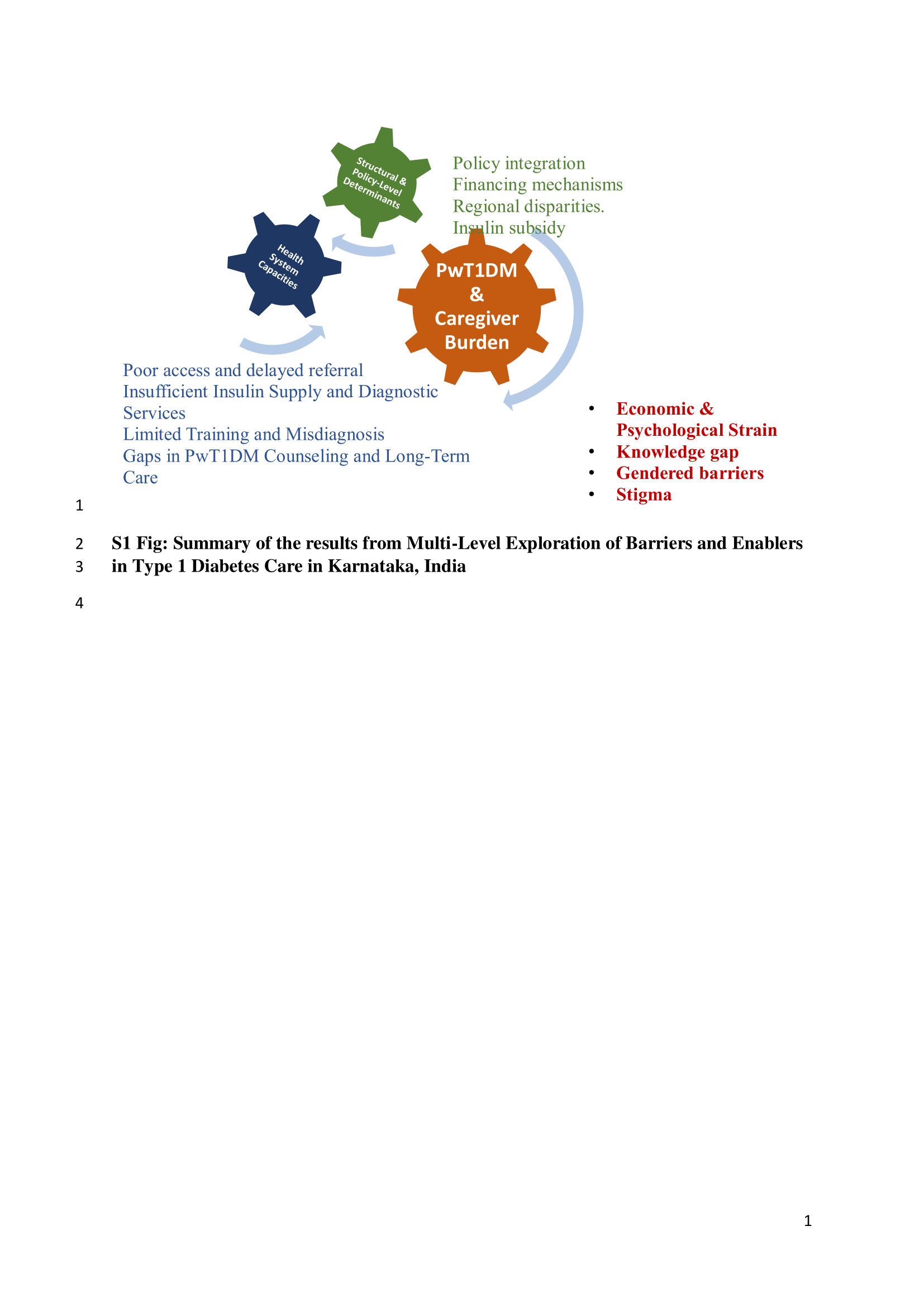

Supplement: S1 Fig — (TIFF) [file pgph.0005129.s001.tiff]
