# Supplementary material for: Invisible inequities in type I diabetes care in India: A multi-stakeholder qualitative study from Karnataka
Source: PLOS Glob Public Health. 2025 Sep 12;5(9):e0005129. doi: 10.1371/journal.pgph.0005129 (PMC12431490; doi:10.1371/journal.pgph.0005129)
Supplement: S1 Appendix — (DOCX) [file pgph.0005129.s002.docx]

**S 1_Appendix - Guide for IDI and FGD**

**Interview Guide for Health care providers’/ Policy makers**

Name of Interviewer: Date of Interview: Time : Place : Job Title :

**Introduction:** Good morning/ afternoon

My name is [Name]. We want to understand the current policies, strategies and interventions in place for T1DM management and to identify the barriers and opportunities to strengthen the infrastructure and resources allocated for T1DM management in Karnataka state. Could you please answer these questions for us to gain some insights? We hope to put across recommendations on improving the situation of management of T1DM in Karnataka. We will be taking a few minutes of your precious time not more than 45 minutes.

Thank you very much for agreeing to take part in this interview. I will now switch on the audio recorder. Remember that you do not have to answer questions you don’t feel comfortable with and can end the interview at any time.

- Can you briefly introduce yourself and explain your role and responsibilities concerning NCD diseases, specifically focusing on T1DM? How long have you been in this position?
- What current interventions and strategies are in place for T1DM in your district?
- Can you describe the policies, guidelines, and plans concerning T1DM in India/Karnataka/District/City? How can one access them?
- Are there challenges in achieving NPCDCS uptake targets for T1DM? If so, can you explain why?
- How do updates or changes in national policies get disseminated at the district level? Is this communication effective?
- Regarding resource allocation:
  - What factors influence the NCDs and NPCDCS budget in your district?
  - Can you discuss the allocation and utilization of resources for T1DM prevention and management?
- Who are the main stakeholders involved in T1DM screening and management? How do they collaborate?
- Can you elaborate on the data management systems for T1DM? How is data shared, protected, and used for improvements?
- What challenges and barriers impact T1DM screening and management? Do you have recommendations to enhance the program's effectiveness?
- How does the district engage communities and schools to raise T1DM awareness, and how do you assess their success?
- Could you describe the healthcare infrastructure available for pediatric T1DM care? Are there plans to enhance it?
- How are parents, caregivers, and educational institutions educated about T1DM?
- Can you elaborate on the quality-of-care assessment for T1DM? How do collaborations with the private sector or NGOs assist in this?
- Are there technological or tele medical solutions employed for T1DM in remote areas?
- How is the mental well-being of children with T1DM addressed? Are there integrated mental health support initiatives?
- Are you familiar with global best practices for T1DM management? How do you stay updated on new developments?
- What are your views on pediatric T1DM screening benefits and risks?
- Lastly, can you touch upon long-term monitoring, psychosocial support, and feedback mechanisms in place for children with T1DM?

Thanks for your kind participation

**Interview guide for Parents/ care givers**

Name of Interviewer Date of Interview

Time Started Time Finished Job Title

**Introduction:**

Good morning/ good afternoon

My name is [Name]. Thank you for consenting to be interviewed. We are here to discuss about the T1DM and your experience in seeking treatment and management of this health condition for your child / ward.

1. Name of the respondent:
2. Age:
3. State:
4. District:
5. Taluka:
6. Place of residence (Urban/rural):
7. Education:
8. Occupation:
9. Total number of family members:
10. Type of Family (Nuclear=1, Joint=2, three generation=3, others=4):
11. Total monthly income of family:
12. Total per capita income (to be calculated by the interviewer):

- Were you aware of T1DM before your child was diagnosed?
- Do you know how this condition occurs?
- What was your reaction when you were informed about the diagnosis for the first time?
- What was the age of the child?
- Is there any other member in the family who has type 1 diabetes?
- Did your child’s diagnosis have an impact on your life? If so, how?
- Has your child’s diagnosis had an impact on others in the family?
- Has the diagnosis has had an impact on your child’s life? If so, in what ways has it impacted them?
- How is your child coping with the current condition?
- What is your role in managing the health condition of your child?
- Were you counselled / educated about the health condition during the time of diagnosis?
- What are the difficulties you faced in accessing health care or providing care to your child?
- How satisfied are you with the care provided by the health care team?
- How convenient is it for you to access healthcare facilities/ specialties/medications?
- What are the difficulties faced during the administration of Insulin?
- How do you store insulin at home?
- How is the insulin stored in the school?
- Are you aware about the dietary restrictions of your child?
- If there are any difficulties or complications whom do you approach?
- Has your involvement changed over time? If yes, in what way
- What changes or challenges have you experienced in this role?
- Do you have any concerns/fears? What contributes to these fears?
- Have these changed as your child emerges/emerged into adulthood?
- Was the health care team helpful in facilitating the transition in adulthood? If so, what did you find to be particularly helpful? If not, how could you or your child have been better supported?
- How have you (and your child) adjusted to this/these change(s)?
- Has the adjustment been challenging or relatively easy? Are there any strategies that you have found helpful in adjusting to these changes?
- Do you feel that there are adequate resources and supports for parents/guardians of young adults with diabetes?
- What support do you expect from the government/healthcare providers?
- What changes do you expect from the government / doctors for better management of your child’s condition?

Thanks for your kind participation

**Interview guide for PwT1DM**

Name of Interviewer

Date of Interview

Time Started

Time Finished

Location

Job Title

**Introduction:**

Good morning/ good afternoon

My name is [Name]. Thank you for consenting to be interviewed. We are here to discuss about the T1DM and your experience in seeking treatment and management of this health condition.

1. Socio-demographics
   1. Name:
   2. Gender:
   3. Age:
   4. District:
   5. Name of Taluk:
   6. Place of residence (Urban / Rural):
   7. Current education/occupation:
   8. School (Govt. / Private):
2. Socio-economic status
   1. Qualification of
      1. Father:
      2. Mother:
      3. Guardian (if any):
   2. Occupation of
      1. Father:
      2. Mother:
      3. Guardian (if any):
   3. Annual income of the family
   4. House (Own / Rented)
3. Family Background
   1. Total number of family members
   2. Type of family (Nuclear / Joint)
   3. No. of siblings
   4. Family History of Diabetes

- How old were you when you were diagnosed?
- What were some of your symptoms?
- Do you know what health condition you are being treated for?
- What do you know about diabetes?
- Can you tell us about the Complications of this condition?
- How do you manage your diabetes?
- Did you ever feel the need to hide it from people?

## Were you worried about managing your diabetes at school?

## Has diabetes ever affected your education?

## How do you manage hypoglycemia?

## What’s the hardest thing about having diabetes?

## Has diabetes have an impact on your daily life? If yes, how

## Do you play any outdoor games? If no, why

## How do you cope up with your current health condition?

- How many times do you administer insulin in a day?
- Self-administration / assisted administration
- Device used (Pen / Syringe)
- Disposal of syringes
- Storage of Insulin in school/workplace / during travel
- The issue in storage during electricity interruptions
- Ever hospitalized for complications
- Are you aware of what foods you need to eat and what are the foods to avoid?
- Challenges
  - Accessibility of Health Care Services
  - Affordability
  - Needle anxiety
  - Society / School / Workplace
  - Stigma (Society / School / Workplace). Ways to handle
  - Impact on family
- Do you have Support from family, relatives, friends, at school? In what way
- What is your expectation from the government / doctors for better management?

Thanks for your kind participation

**FGD guide**

Name of moderator Date of FGD

Time Started Time Finished

**Introduction:**

Good morning/ good afternoon

My name is [name]. Thank you for consenting to be part of this Focus group discussion. I am here to discuss about the T1DM and your experience in seeking treatment and management of this health condition for your child/ yourself.

1. Can you tell me about when your child was first diagnosed with type 1 diabetes?

- How old were they?
- Did you have any prior experience with type 1 diabetes?
- Did your child’s diagnosis have an impact on your life? If so, how?
- Did your child’s diagnosis have an impact on everyday life in the household? Can you tell me about that?
- Did it impact the family dynamic?
- As your child’s diagnosis had an impact on others in the family?
- Do you perceive that the diabetes diagnosis has had an impact on your child’s life? If so, in what ways has it impacted them?
- Have you noticed any differences in the impact of diabetes on their life as they’ve gotten older?
- How do you see diabetes impacting them currently?

1. How would you describe your involvement in the management of your child’s diabetes?

- What illness-management tasks do you routinely assist with?
- How do you perceive your child’s diabetes self-management practices?
- Has your involvement changed over time?
- Were you counselled / educated about the health condition during the time of diagnosis?
- How is your child coping with the current condition?
- What are the difficulties you faced in accessing health care or providing care to your child?
- What is your role in managing the health condition of your child?
- How satisfied are you with the care provided by the health care team?
- How convenient is it for you to access healthcare facilities/ specialties/medications?
- Has your involvement changed over time? If yes, in what way
- What changes or challenges have you experienced in this role?

1. Drawing on your own experiences, can you tell me what is it like to be a parent/guardian of a young adult who is living with type 1 diabetes?

- What changes or challenges have you experienced in this role?
- Do you have any concerns/fears? What contributes to these fears?
- Have these changed as your child emerges/emerged into adulthood?
- What helps you to manage your concerns/fears?
- Do you feel that there are adequate resources and supports for parents/guardians of young adults with diabetes? If so, what are some of the resources and supports that you have found particularly helpful? If not, what resources and supports would you like to have access to (e.g. information, support groups, etc.)?

1. Informed by these experiences, and with the benefit of hindsight, what are your expectation from the government and health system?

- Do you feel that there are adequate resources and supports for parents/guardians of young adults with diabetes?
- What support do you expect from the government/healthcare providers?
- What changes do you expect from the government / doctors for better management of your child’s condition?

1. Is there anything we didn’t get a chance to discuss that you think would be important for us to know?

Thanks for your kind participation
