# Supplementary material for: Invisible inequities in type I diabetes care in India: A multi-stakeholder qualitative study from Karnataka
Source: PLOS Glob Public Health. 2025 Sep 12;5(9):e0005129. doi: 10.1371/journal.pgph.0005129 (PMC12431490; doi:10.1371/journal.pgph.0005129)
Supplement: S2 Appendix — (DOCX) [file pgph.0005129.s003.docx]

**S2_Appendix - Code Book**

**Code Book for People with Type-1 Diabetes Mellitus (PwT1DM)**

**Part 1 – Socio-Demographic Codebook**

**Parent Node: Demographic Information**
Child Node:

- Gender – Self-reported gender of the participant.
- Age – Age in years at the time of the interview.
- Education Level – Highest educational level attained.
- Schooling Type – Type of school attended (government or private).

**Parent Node: Occupational and Economic Status**
Child Node:

- Occupation – Current work or employment status of participant.
- Parental Occupation – Occupation(s) of participant’s parents/guardians.
- Annual Family Income – Total reported yearly household income.

**Parent Node: Family Background**
Child Node:

- Parental Education – Highest education level of father, mother, or guardian.
- Family Type – Household structure (nuclear, joint, extended).
- Number of Siblings – Count of participant’s brothers and sisters.

**Parent Node: Health-related Family History**
Child Node:

- Family History of Diabetes – Whether immediate family members have diabetes.

**Part 2 – Qualitative Thematic Codebook**

**Parent Node: Health System and Policy Gaps**
Child Node:

- Limited Policy Integration and Financial Protection – Lack of prioritization of T1DM in national/state health policies and inadequate funding support.
- Limited Training and Misdiagnosis Risks – Insufficient training among healthcare providers leading to delayed or incorrect diagnosis.
- Inadequate Supply Chain and Resource Availability – Gaps in insulin supply, monitoring tools, and related infrastructure.

**Parent Node: Provider and Workforce Perspectives**
Child Node:

- Awareness and Knowledge Gaps – Provider-level misconceptions or lack of T1DM-specific expertise.
- Workload and Systemic Constraints – High patient loads and limited time/resources for T1DM care.

**Parent Node: Patient and Caregiver Experiences**
Child Node:

- Financial Burden of Care – Out-of-pocket expenses and challenges affording insulin and supplies.
- Access Barriers – Travel distance, appointment availability, and bureaucratic hurdles in accessing services.
- Gendered Barriers and Social Stigma – Gender-based discrimination and societal stigma impacting care-seeking.

**Parent Node: Potential Solutions and Interventions**
Child Node:

- Policy Reforms – Suggested strategies for integrating T1DM into health programs.
- Capacity Building – Training for providers and awareness campaigns for communities.
- Financial Protection Mechanisms – Proposals for insurance coverage and subsidies.

**Code Book for Caregivers**

**Part 1 – Socio-Demographic Codebook**

**Parent Node:** Demographic Information
**Child Node:**

- **Gender** – Self-reported gender of the participant.
- **Age** – Age in years at the time of the interview.
- **Professional Role** – Designation or role within the healthcare system (e.g., endocrinologist, nurse, program manager).
- **Years of Experience** – Total number of years working in healthcare.
- **Sector** – Employment sector (government, private, or NGO).
- **Geographical Location** – State or district where the participant is based.

**Part 2 – Qualitative Thematic Codebook**

**Parent Node:** Policy and Governance
**Child Node:**

- **Policy Gaps** – Lack of specific inclusion of T1DM in national/state health strategies.
- **Programmatic Integration** – Weak or absent integration of T1DM into existing NCD programs.
- **Data Systems** – Inadequate or inconsistent data collection and reporting mechanisms.

**Parent Node:** Healthcare Service Delivery
**Child Node:**

- **Human Resource Constraints** – Shortage of trained professionals for T1DM care.
- **Infrastructure Gaps** – Limited availability of diagnostic and monitoring facilities.
- **Referral Pathways** – Weak linkages between different levels of care.

**Parent Node:** Access to Care
**Child Node:**

- **Cost Barriers** – Out-of-pocket expenses for insulin, strips, and devices.
- **Supply Chain Issues** – Irregular availability of essential medicines and supplies.
- **Geographical Barriers** – Distance and transportation challenges to access specialized care.

**Parent Node:** Community and Patient Factors
**Child Node:**

- **Awareness and Knowledge** – Low public awareness about T1DM symptoms and management.
- **Cultural Beliefs and Stigma** – Social attitudes that hinder care-seeking or adherence.
- **Gender-Based Constraints** – Gender norms affecting access to timely diagnosis and treatment.

**Parent Node:** Recommendations
**Child Node:**

- **Policy Advocacy** – Inclusion of T1DM in government health priorities and schemes.
- **Capacity Building** – Training healthcare providers for better diagnosis and management.
- **Community Engagement** – Awareness drives, peer support, and stigma reduction strategies.

**Code Book for Health Care Providers**

**Part 1 – Socio-Demographic Codebook**

**Parent Node:** Demographic Information
**Child Node:**

- **Gender** – Self-reported gender of the participant.
- **Age** – Age in years at the time of the interview.
- **Professional Role** – Designation or role within the healthcare system (e.g., endocrinologist, nurse, program manager).
- **Years of Experience** – Total number of years working in healthcare.
- **Sector** – Employment sector (government, private, or NGO).
- **Geographical Location** – State or district where the participant is based.

**Part 2 – Qualitative Thematic Codebook**

**Parent Node:** Policy and System-Level Barriers
**Child Node:**

- **Limited Policy Integration** – Absence or inadequate inclusion of T1DM in national/state health policy frameworks.
- **Funding Gaps** – Lack of structured financing for insulin, monitoring supplies, and other T1DM needs.
- **Data Gaps** – Inadequate data collection or under-reporting of T1DM cases in health system records.

**Parent Node:** Service Delivery Challenges
**Child Node:**

- **Workforce Shortages** – Lack of trained endocrinologists, diabetes educators, or nutritionists.
- **Infrastructure Limitations** – Insufficient facilities for diagnosis, follow-up, and patient education.
- **Referral Gaps** – Weak coordination between primary, secondary, and tertiary care levels.

**Parent Node:** Patient and Community-Level Barriers
**Child Node:**

- **Awareness Gaps** – Low awareness among the public about T1DM symptoms and management.
- **Social Stigma** – Negative attitudes and discrimination towards people living with T1DM.
- **Gendered Barriers** – Gender-specific challenges in access to care and adherence.

**Parent Node:** Proposed Solutions and Recommendations
**Child Node:**

- **Policy Advocacy** – Need for inclusion of T1DM in NCD programs and state health agendas.
- **Capacity Building** – Training for healthcare workers on T1DM management.
- **Community Engagement** – Public awareness campaigns to reduce stigma and promote early diagnosis.

**Code Book for Senior Health Officials and Policymakers**

**Part 1 – Socio-Demographic Codebook**

**Parent Node:** Demographic Information
**Child Nodes:**

- **Gender** – Self-reported gender of the participant.
- **Age** – Age in years at the time of the interview.
- **Professional Role** – Designation or role within the government/public health system (e.g., Reproductive and Child Health Officer).
- **Years of Experience** – Total number of years working in the health sector.
- **Current Position Tenure** – Number of months/years in the current role.
- **Sector** – Government sector position (district, taluka, or state-level role).
- **Geographical Location** – State and district where the participant is based.

**Part 2 – Qualitative Thematic Codebook**

**Parent Node: Policy and System-Level Barriers**

**Child Nodes:**

- **Absence of T1DM-Specific Program** – No structured protocol or program for paediatric T1DM management.
- **Fragmented Service Delivery** – T1DM care occurring only when cases are diagnosed; not systemized or included in vertical programs.
- **Data Gaps** – No dedicated statistics for paediatric T1DM cases at district level; focus remains on communicable and vaccine-preventable diseases.
- **Policy Invisibility** – T1DM not integrated into current child health or NCD policies at district level.
- **Infrastructure Enhancement Plans Missing** – No concrete district-level plans to strengthen paediatric T1DM facilities.

**Parent Node: Service Delivery Challenges**

**Child Nodes:**

- **Detection Limitations at Primary Level** – PHCs and CHCs lack capacity for paediatric blood sampling and diagnosis.
- **Skilled Workforce Shortages** – Shortage of staff trained to perform paediatric venepuncture and manage T1DM.
- **Limited Referral System Strength** – Reliance on higher centres like district hospitals and Bangalore; no structured referral pathway.
- **Private Sector Non-Cooperation** – Private schools and facilities often do not participate in government health initiatives.

**Parent Node: Patient and Community-Level Barriers**

**Child Nodes:**

- **Low Public Awareness** – Community unfamiliar with paediatric T1DM symptoms and risks.
- **Low Literacy Impact** – Education level influences disease acceptance; delayed acknowledgment after diagnosis.
- **Stigma and Low Acceptance** – Families reluctant to accept T1DM diagnosis, requiring repeated counselling.
- **Economic Barriers** – Families in lower socioeconomic strata face difficulties in ensuring nutrition, school attendance, and supervision.

**Parent Node: Proposed Solutions and Recommendations**

**Child Nodes:**

- **Government Program Inclusion** – Integration of T1DM into existing child health and NCD frameworks.
- **Public–Private Partnerships** – Engage private schools and organizations for awareness and screening programs.
- **Capacity Building** – Train PHC/CHC staff for paediatric diagnosis and insulin administration.
- **Community Awareness Campaigns** – Use ASHA workers, Anganawadi meetings, and mothers’ meetings to spread awareness.
- **Peer and Parent Counselling** – Promote one-to-one counselling rather than mass awareness alone.
- **Early Detection Focus** – Encourage facility-based detection with strengthened monitoring infrastructure.
