# Supplementary material for: Invisible inequities in type I diabetes care in India: A multi-stakeholder qualitative study from Karnataka
Source: PLOS Glob Public Health. 2025 Sep 12;5(9):e0005129. doi: 10.1371/journal.pgph.0005129 (PMC12431490; doi:10.1371/journal.pgph.0005129)
Supplement: S1 Text — (DOCX) [file pgph.0005129.s004.docx]

# S1 Text - Excerpts from qualitative transcripts

This file presents selected anonymised quotations from participants (People with Type 1 Diabetes Mellitus, caregivers, healthcare providers, and policymakers). These excerpts illustrate key themes discussed in the article while preserving confidentiality.

## Theme 1: Structural and Policy-Level Determinants

- “The Government hospitals provide insulin only in limited stock, and there is no guarantee it will be available when we need it. We have to buy it from private pharmacies, which cost almost half of my monthly income.” (Caregiver, Rural, Female)
- “We sometimes reduce the insulin dose to make it last longer because we can’t always afford to buy more.” (Caregiver, Urban, Male)

## Theme 2: Health System Capacities and Service Delivery Constraints

- “We receive insulin in unpredictable batches. When supplies run out, PwT1DM has to buy them from outside, which many cannot afford. We need a better system to ensure a continuous and predictable stock of insulin.” (Physician, District Hospital)
- “We focus on immediate treatment rather than long-term management. Without regular monitoring tools like HbA1c tests, we are treating diabetes blindly, which leads to complications.” (Government Health Official)
- “For my child’s diabetes, we must travel to the city. The cost of transport is almost as much as the medicine itself. Sometimes we skip appointments because we just can’t afford to go.” (Mother, Rural Caregiver)
- “By the time a child reaches a specialised centre, their condition has worsened.” (Doctor, Rural PHC)

## Theme 3: PwT1DM and Caregiver Burden

- “I had to leave my job because my son needs constant care. Now, we are struggling financially, and sometimes I worry about whether we will have enough money to buy his next insulin dose.” (Mother, Caregiver)
- “I feel guilty every time my parents struggle to buy my medicines. Sometimes, I think it would be easier if I weren’t sick.” (Boy, 14 years)
- “My family told me not to talk about my condition outside because they were afraid that I wouldn’t find a husband.” (Adolescent Girl, PwT1DM)
- “During exams, I needed to take breaks to check my sugar levels, but my teachers thought I was making excuses.” (College Student, PwT1DM)

## Theme 4: Health care Provider Perspectives

- “In medical school, we learned a lot about T2DM, but T1DM was barely covered. Many of us rely on general knowledge, which may not always be enough. This may lead to misdiagnoses, especially in young children.” (Physician, Government Hospital)
- “Most of the time, we prescribe insulin and move on to the next patient. We won’t have time to provide detailed counselling on diet and lifestyle.” (Physician, Government Hospital)
- “Many patients believe insulin injections are the last option and hence hesitate to start therapy.” (Healthcare Provider)
- “In urban centres, patients have access to dieticians, diabetes educators, and multidisciplinary teams. However, in rural areas, primary care doctors and nurses are often expected to perform a wide range of tasks without additional training.” (Endocrinologist, Private Hospital)
- “We often see children come with severe complications because they can’t afford insulin or didn’t understand the importance of daily injections. It’s heartbreaking to see such situations.” (Paediatric Endocrinologist)
- “We are overwhelmed. We hardly have time to explain insulin use properly, let alone the emotional stress these families face.” (Government HCP)

## Theme 5: Stakeholders’ Perspectives on Solutions

- “We have seen successful state-level programs making insulin affordable, but we need a national-level strategy to ensure uniform access.” (Policymaker)
- “We cannot expect rural patients to travel long distances to tertiary hospitals for routine diabetes care. Strengthening PHCs is the only way to ensure continuity of treatment.” (Policy Stakeholder)
- “Telemedicine can help bridge the gap for patients in remote areas who struggle to access specialised care.” (Healthcare Professional)
- “Talking to others who have T1DM helped me realise I'm not alone.” (Young Participant, PwT1DM)
- “Before joining [peer group], we were overwhelmed and confused, but now we feel more confident in managing our child’s condition.” (Caregiver, Parent)
